# Supplementary material for: Fast and efficient purification of SARS-CoV-2 RNA dependent RNA polymerase complex expressed in Escherichia coli
Source: PLoS One. 2021 Apr 29;16(4):e0250610. doi: 10.1371/journal.pone.0250610 (PMC8084133; doi:10.1371/journal.pone.0250610)
Supplement: S1 File — (PDF) [file pone.0250610.s001.pdf]

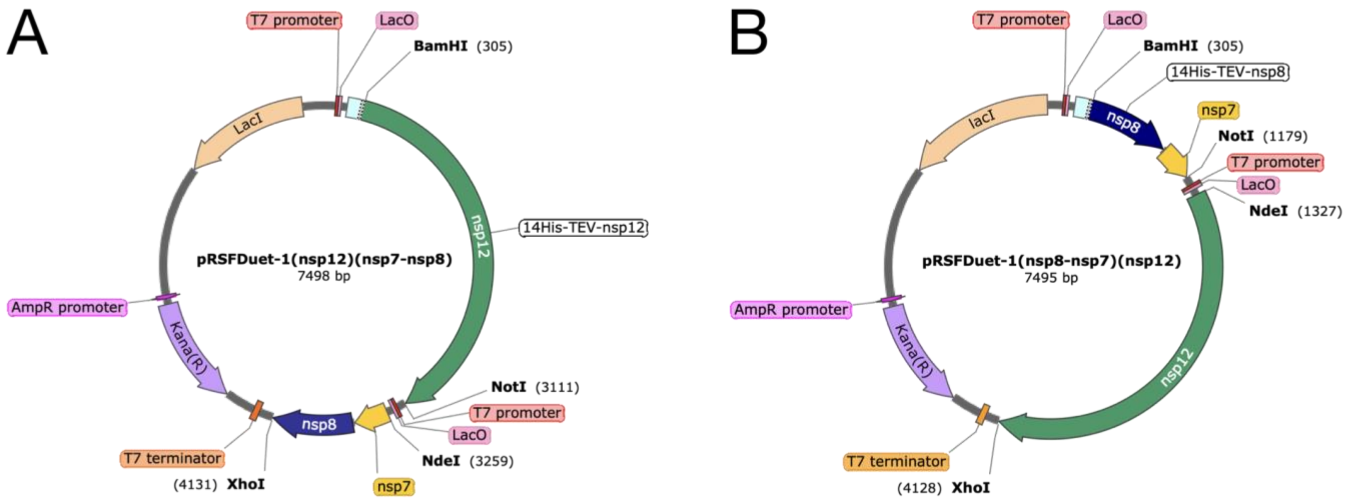

S1 Fig. Recombinant expression plasmid maps

Plasmid map of the pRSFDuet-1(14His-nsp12)(nsp7/nsp8) **(A)** and pRSFDuet-1(14His-nsp8/nsp7)(nsp12) **(B)** used for recombinant expression of the SARS-CoV-2 RdRp complex in *E. coli*.

```
1 gctatcatgc cataccgcga aaggttttgc gccattcgat ggtgtccggg atctcgacgc tctcccttat gcgactcctg cattaggaaa ttaatacgac
101 tcactatagg ggaattgtga gcggataaca attcccctgt agaaataatt ttgtttaact ttaataagga gatataccAT GGGCAAGCAT CACCATCATT
201 CAGGCCATCA CCATACCGGA CACCACCATC ATTCAGGCAG TCATCACCAT ACTAGTAGCA GCGCTAGCAC CGGCGAGAAC CTGTATTTTC AAGGGACGGG
301 GGATGGATCC GCAATTGCAA GCGAATTTAG CAGCCTGCCG AGCTATGCAG CATTTGCAAC CGCACAAGAG GCCTATGAAC AGGCAGTTGC CAATGGTGAT
401 AGCGAAGTTG TTCTGAAAAA ACTGAAAAAG TCACTGAACG TGGCCAAATC CGAATTTGAT CGTGATGCAG CAATGCAGCG TAAACTGGAA AAAATGGCAG
501 ATCAGGCAAT GACCCAGATG TATAAACAGG CACGTAGCGA AGATAAACGT GCAAAAGTTA CCAGCGCAAT GCAGACCATG CTGTTTACCA TGCTGCGCAA
601 ACTGGATAAT GATGCCCTGA ACAACATTAT CAATAATGCC CGTGATGGTT GTGTTCCGCT GAACATTATT CCGCTGACCA CCGCAGCAAA ACTGATGGTG
701 GTTATTCCGG ATTACAACAC CTATAAAAAC ACCTGTGATG GCACCACCTT TACCTATGCA AGCGCACTGT GGGAAATTCA GCAGGTTGTT GATGCAGATA
801 GCAAAATTGT TCAGCTGAGC GAAATTAGCA TGGATAATAG CCCGAATCTG GCATGGCCTC TGATTGTTAC CGCACTGCGT GCAAATAGCG CAGTTAAACT
901 GCAGTAAaat aaggagatat acATGAGCAA AATGAGTGAT GTTAAATGTA CCAGCGTTGT TCTGCTGAGC GTTCTGCAGC AGCTGCGTGT TGAAAGCAGC
1001 AGCAAACTGT GGGCACAGTG TGTTCAGCTG CATAATGATA TTCTGCTGGC AAAAGATACC ACCGAAGCCT TTGAAAAAAT GGTTAGCCTG CTGAGTGTGC
1101 TGCTGAGCAT GCAGGGTGCA GTTGATATTA ACAAACTGTG TGAAGAGATG CTGGATAATC GTGCAACCCT GCAGTAAgcg gccgcataat gcttaagtcg
1201 aacagaaagt aatcgtattg tacacggccg cataatcgaa attaatacga ctcactatag gggaattgtg agcggataac aattccccat cttagtatat
1301 tagttaagta taagaaggag atatacatAT GAGCGCAGAT GCACAGAGCT TTCTGAATCG TGTTTGTGGT GTTAGCGCAG CACGTCTGAC CCCGTGTGGC
1401 ACCGGCACCA GCACCGATGT TGTTTATCGT GCATTTGATA TCTACAACGA TAAAGTTGCC GGTTTCGCCA AATTTCTGAA AACCAATTGT TGCCGCTTCC
1501 AAGAGAAAGA TGAAGATGAT AATCTGATCG ACAGCTACTT TGTGGTTAAA CGTCATACCT TTAGCAACTA TCAGCACGAG GAAACCATTT ACAATCTGCT
1601 GAAAGATTGT CCGGCAGTTG CCAAACATGA TTTTTTCAAA TTTCGCATCG ATGGCGATAT GGTTCCGCAT ATTAGCCGTC AGCGTCTGAC CAAATATACC
1701 ATGGCAGATC TGGTTTATGC ACTGCGTCAT TTTGATGAAG GTAATTGCGA TACCCTGAAA GAAATTCTGG TGACCTATAA TTGTTGCGAC GATGACTACT
1801 TCAACAAGAA AGATTGGTAT GATTTCGTGG AAAACCCGGA TATTCTGCGT GTTTATGCAA ATCTGGGTGA ACGTGTTCGT CAGGCACTGC TGAAAACCGT
1901 TCAGTTTTGT GATGCAATGC GTAATGCAGG TATTGTTGGT GTTCTGACCC TGGATAATCA GGATCTGAAT GGTAATTGGT ACGATTTTGG CGATTTTATT
2001 CAGACCACAC CTGGTAGCGG TGTTCCGGTT GTTGATAGCT ATTATAGCCT GCTGATGCCG ATTCTGACAC TGACCCGTGC ACTGACCGCA GAAAGCCATG
2101 TTGATACCGA TCTGACAAAA CCGTACATTA AATGGGACCT GCTGAAATAT GATTTTACCG AAGAACGCCT GAAACTGTTC GATCGCTATT TCAAATATTG
2201 GGACCAGACC TATCATCCGA ATTGTGTTAA TTGTCTGGAT GATCGTTGTA TTCTGCATTG CGCCAATTTT AACGTTCTGT TTAGCACCGT TTTTCCGCCT
2301 ACCAGCTTTG GTCCGCTGGT TCGTAAAATC TTTGTTGATG GTGTGCCGTT TGTTGTTAGC ACCGGTTATC ATTTTCGTGA ACTGGGTGTT GTTCATAACC
2401 AGGATGTTAA TCTGCATAGC AGCCGTCTGA GCTTTAAAGA ACTGCTGGTG TATGCAGCAG ATCCGGCAAT GCATGCAGCA AGCGGTAATC TGCTGCTGGA
2501 TAAACGTACC ACCTGTTTTA GCGTTGCAGC ACTGACCAAT AATGTTGCAT TTCAGACCGT TAAACCGGGT AATTTCAACA AGGATTTCTA TGATTTTGCC
2601 GTGAGCAAGG GCTTTTTCAA AGAAGGTAGC AGCGTTGAAC TGAAGCATTT TTTCTTTGCA CAGGATGGTA ATGCAGCCAT CAGCGATTAT GATTATTATC
2701 GCTATAATCT GCCGACCATG TGTGATATTC GTCAGCTGCT GTTTGTTGTG GAAGTTGTGG ATAAATACTT CGATTGCTAT GATGGCGGTT GCATTAACGC
2801 AAATCAGGTG ATTGTTAATA ACCTGGACAA AAGCGCAGGT TTCCCGTTTA ACAAATGGGG TAAAGCCCGT CTGTATTATG ATAGCATGAG CTATGAAGAT
2901 CAGGATGCAC TGTTTGCATA TACCAAACGT AATGTGATTC CGACCATTAC GCAGATGAAT CTGAAATACG CAATTAGCGC AAAAAATCGT GCACGTACCG
3001 TTGCCGGTGT TAGCATTTGT AGCACCATGA CCAATCGTCA GTTTCATCAG AAACTGCTGA AGAGTATTGC AGCAACCCGT GGTGCAACCG TTGTTATTGG
3101 TACAAGCAAA TTCTATGGTG GCTGGCATAA TATGCTTAAG ACCGTGTATT CAGATGTGGA AAATCCGCAT CTGATGGGTT GGGATTATCC GAAATGTGAT
3201 CGTGCAATGC CGAATATGCT GCGTATTATG GCAAGCCTGG TTCTGGCACG TAAACATACC ACATGTTGCA GCCTGAGCCA TCGTTTTTAT CGTCTGGCAA
3301 ATGAATGTGC ACAGGTTCTG AGCGAAATGG TTATGTGTGG TGGTAGCCTG TATGTTAAAC CTGGTGGCAC CAGCTCAGGT GATGCAACCA CCGCCTATGC
3401 AAATAGCGTT TTTAACATTT GTCAGGCCGT TACCGCCAAT GTTAATGCCC TGCTGAGTAC CGATGGCAAC AAAATTGCAG ATAAATATGT GCGCAATCTG
3501 CAGCATCGTC TGTATGAATG TCTGTATCGT AATCGTGATG TGGATACCGA CTTTGTGAAT GAGTTTTATG CCTATCTGCG CAAACACTTC AGCATGATGA
3601 TTCTGAGTGA TGATGCCGTT GTTTGCTTTA ATAGCACCTA TGCAAGCCAA GGTCTGGTTG CAAGCATCAA AAACTTTAAA AGCGTGCTGT ACTACCAGAA
3701 CAATGTGTTT ATGAGCGAAG CAAAATGTTG GACCGAAACC GACCTGACCA AAGGTCCGCA TGAATTTTGT AGCCAGCATA CCATGCTGGT TAAACAGGGT
3801 GATGATTATG TTTATCTGCC GTATCCTGAT CCGAGCCGTA TTTTAGGTGC AGGTTGTTTT GTGGATGACA TCGTTAAAAC CGATGGTACG CTGATGATTG
3901 AACGTTTTGT TAGCCTGGCA ATTGATGCAT ATCCGCTGAC CAAACATCCG AATCAAGAAT ATGCAGATGT GTTCCATCTG TACCTGCAGT ATATTCGTAA
4001 ACTGCATGAT GAACTGACAG GTCACATGCT GGATATGTAT AGCGTTATGC TGACCAATGA TAATACCAGC CGTTATTGGG AACCCGAATT TTATGAAGCA
4101 ATGTATACAC CGCATACCGT GCTGTAActc gagtctggta aagaaaccgc tgctgcgaaa tttgaacgcc agcacatgga ctcgtctact agcgcagctt
4201 aattaaccta ggctgctgcc accgctgagc aataactagc ataacccctt ggggcctcta aacgggtctt gaggggtttt ttgctgaaac ctcaggcatt
4301 tgagaagcac acggtcacac tgcttccggt agtcaataaa ccggtaaacc agcaatagac ataagcggct atttaacgac cctgccctga accgacgaca
4401 agctgacgac cgggtctccg caagtggcac ttttcgggga aatgtgcgcg gaacccctat ttgtttattt ttctaaatac attcaaatat gtatccgctc
4501 atgaattaat tcTTAGAAAA ACTCATCGAG CATCAAATGA AACTGCAATT TATTCATATC AGGATTATCA ATACCATATT TTTGAAAAAG CCGTTTCTGT
4601 AATGAAGGAG AAAACTCACC GAGGCAGTTC CATAGGATGG CAAGATCCTG GTATCGGTCT GCGATTCCGA CTCGTCCAAC ATCAATACAA CCTATTAATT
4701 TCCCCTCGTC AAAAATAAGG TTATCAAGTG AGAAATCACC ATGAGTGACG ACTGAATCCG GTGAGAATGG CAAAAGTTTA TGCATTTCTT TCCAGACTTG
4801 TTCAACAGGC CAGCCATTAC GCTCGTCATC AAAATCACTC GCATCAACCA AACCGTTATT CATTCGTGAT TGCGCCTGAG CGAGACGAAA TACGCGGTCG
4901 CTGTTAAAAG GACAATTACA AACAGGAATC GAATGCAACC GGCGCAGGAA CACTGCCAGC GCATCAACAA TATTTTCACC TGAATCAGGA TATTCTTCTA
5001 ATACCTGGAA TGCTGTTTTC CCGGGGATCG CAGTGGTGAG TAACCATGCA TCATCAGGAG TACGGATAAA ATGCTTGATG GTCGGAAGAG GCATAAATTC
5101 CGTCAGCCAG TTTAGTCTGA CCATCTCATC TGTAACATCA TTGGCAACGC TACCTTTGCC ATGTTTCAGA AACAACTCTG GCGCATCGGG CTTCCCATAC
5201 AATCGATAGA TTGTCGCACC TGATTGCCCG ACATTATCGC GAGCCCATTT ATACCCATAT AAATCAGCAT CCATGTTGGA ATTTAATCGC GGCCTAGAGC
5301 AAGACGTTTC CCGTTGAATA TGGCTCATac tcttcctttt tcaatattat tgaagcattt atcagggtta ttgtctcatg agcggataca tatttgaatg
5401 tatttagaaa aataaacaaa taggcatgca gcgctcttcc gcttcctcgc tcactgactc gctacgctcg gtcgttcgac tgcggcgagc ggtgtcagct
5501 cactcaaaag cggtaatacg gttatccaca gaatcagggg ataaagccgg aaagaacatg tgagcaaaaa gcaaagcacc ggaagaagcc aacgccgcag
5601 gcgtttttcc ataggctccg ccccccctgac gagcatcaca aaaatcgacg ctcaagccag aggtggcgaa acccgacagg actataaaga taccaggcgt
5701 ttccccctgg aagctccctc gtgcgctctc ctgttccgac cctgccgctt accggatacc tgtccgcctt tctcccttcg ggaagcgtgg cgctttctca
5801 tagctcacgc tgttggtatc tcagttcggt gtaggtcgtt cgctccaagc tgggctgtgt gcacgaaccc cccgttcagc ccgaccgctg cgccttatcc
5901 ggtaactatc gtcttgagtc caacccggta agacacgact tatcgccact ggcagcagcc attggtaact gatttagagg actttgtctt gaagttatgc
6001 acctgttaag gctaaactga aagaacagat tttggtgagt gcggtcctcc aacccactta ccttggttca aagagttggt agctcagcga accttgagaa
6101 aaccaccgtt ggtagcggtg gtttttcttt atttatgaga tgatgaatca atcggtctat caagtcaacg aacagctatt ccgttactct agatttcagt
6201 gcaatttatc tcttcaaatg tagcacctga agtcagcccc atacgatata agttgtaatt ctcatgttag tcatgccccg cgcccaccgg aaggagctga
6301 ctgggttgaa ggctctcaag ggcatcggtc gagatcccgg tgcctaatga gtgagctaac ttacattaat tgcgttgcgc tcactgcccg ctttccagtc
6401 gggaaacctg tcgtgccagc tgcattaatg aatcggccaa cgcgcgggga gaggcggttt gcgtattggg cgccagggtg gtttttcttt tcaccagtga
6501 gacgggcaac agctgattgc ccttcaccgc ctggccctga gagagttgca gcaagcggtc cacgctggtt tgccccagca ggcgaaaatc ctgtttgatg
6601 gtggttaacg gcgggatata acatgagctg tcttcggtat cgtcgtatcc cactaccgag atgtccgcac caacgcgcag cccggactcg gtaatggcgc
6701 gcattgcgcc cagcgccatc tgatcgttgg caaccagcat cgcagtggga acgatgccct cattcagcat ttgcatggtt tgttgaaaac cggacatggc
6801 actccagtcg ccttcccgtt ccgctatcgg ctgaatttga ttgcgagtga gatatttatg ccagccagcc agacgcagac gcgccgagac agaacttaat
6901 gggcccgcta acagcgcgat ttgctggtga cccaatgcga ccagatgctc cacgcccagt cgcgtaccgt cttcatggga gaaaataata ctgttgatgg
7001 gtgtctggtc agagacatca agaaataacg ccggaacatt agtgcaggca gcttccacag caatggcatc ctggtcatcc agcggatagt taatgatcag
7101 cccactgacg cgttgcgcga gaagattgtg caccgccgct ttacaggctt cgacgccgct tcgttctacc atcgacacca ccacgctggc acccagttga
7201 tcggcgcgag atttaatcgc cgcgacaatt tgcgacggcg cgtgcagggc cagactggag gtggcaacgc caatcagcaa cgactgtttg cccgccagtt
7301 gttgtgccac gcggttggga atgtaattca gctccgccat cgccgcttcc actttttccc gcgttttcgc agaaacgtgg ctggcctggt tcaccacgcg
7401 ggaaacggtc tgataagaga caccggcata ctctgcgaca tcgtataacg ttactggttt cacattcacc accctgaatt gactctcttc cgggc
```

aaaa	T7 promoter
aaaa	LacO
**AAAA**	14-His
AAAA	TEV site
**AAAA**	Nsp8
**AAAA**	Nsp7
**AAAA**	Nsp12
aaaa	T7 terminator
AAAA	Kana(R)
aaaa	AmpR promomoter
aaaa	LacI

S2 Fig. Recombinant expression plasmid sequence

Plasmid sequence of the pRSFDuet-1(14His-nsp8/nsp7)(nsp12) used for recombinant expression of the SARS-CoV-2 RdRp complex in E. coli.

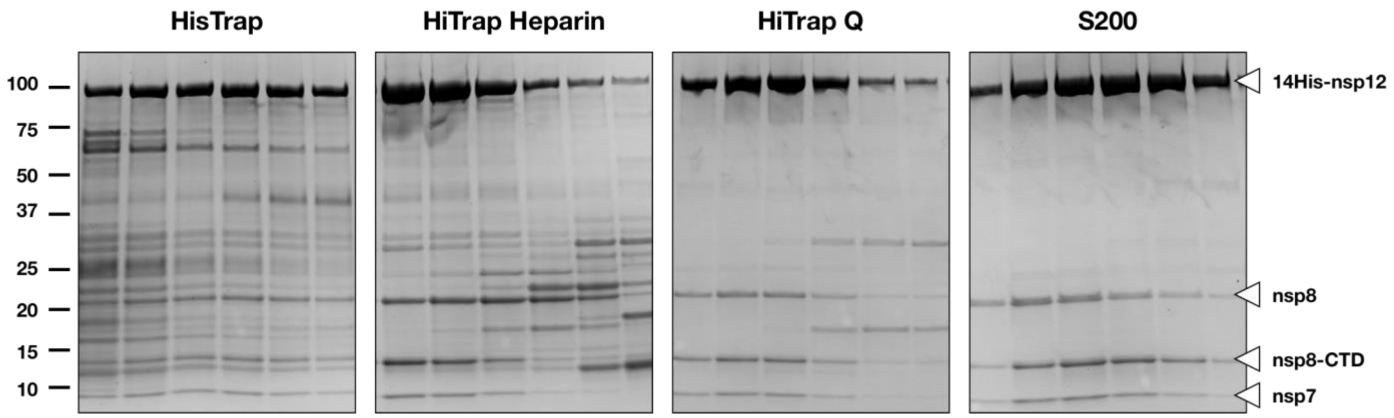

S3 Fig. RdRp production using the pRSFDuet-1(14His-nsp12)(nsp7/nsp8)

Early purification assay of the recombinant RdRp complex expressed in BL21-Star-(DE3)-pRSFDuet-1(nsp12(nsp7-nsp8) cells. Relevant fractions from HisTrap, HiTrap Heparin, HiTrap Q and S200 columns were analysed on SDS-PAGE (4-20%). The extra-band corresponding to nsp8-CTD has been identified through in-gel tryptic digestion coupled with Matrix-Assisted Laser Desorption/Ionization (MALDI-TOF/TOF) analysis.

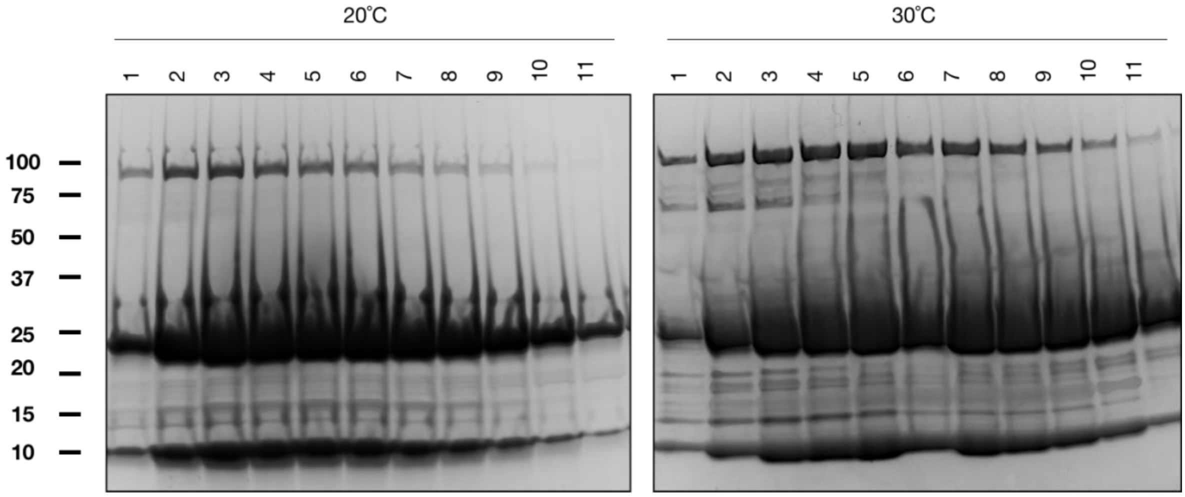

S4 Fig. Influence of post-induction temperatures on HisTrap elution profile.

SDS-PAGE analysis of HisTrap eluted fractions from 20˚C- (left) or 30˚C- (right) induced cells.
