# Supplementary material for: Fast and efficient purification of SARS-CoV-2 RNA dependent RNA polymerase complex expressed in Escherichia coli
Source: PLoS One. 2021 Apr 29;16(4):e0250610. doi: 10.1371/journal.pone.0250610 (PMC8084133; doi:10.1371/journal.pone.0250610)
Supplement: Attachment — Submitted filename: Madru et al..pdf [file pone.0250610.s003.pdf]

The revised manuscript by Madru et al. is greatly improved. The significance of obtaining active RdRp rapidly would facilitate high-throughput screens, and the relative ease of obtaining active protein with one step is commendable. I have a few comments that need addressing, but I do recommend acceptance with some revision.

Because the manuscript does not have page or line numbers, I have copied and pasted where my comments are addressing:

1.The main strategies employed so far for the overexpression of recombinant RdRp consists in expressing and purifying the catalytic nsp12 subunit and the accessory nsp7-nsp8 subunits separately before assembling the complex in vitro (11,22,24,28–30).
-The authors, in their response, cited Dangerfield's 2021 work which also employs a co-expression system, so this claim is false. This paper should be cited within this manuscript. This submitted manuscript's advantage is that the preps can be performed in one day and yield active enzymes. I appreciate the authors did initiate these studies before Dangerfield's paper was published. Therefore, they could state while their manuscript was in review, another group published a co-expression system but one that uses at least 4 for five steps.

2. Our fast, single-day purification protocol results in a stable and active complex that can be used in most protein biochemistry laboratories for drug screening, as well as for routine functional and structural studies
-This approach of a one-day prep shows the protein is active, but they did not show the prep to be suitable for structural studies. Please remove that claim.

3. Depending on the applications, the N-terminal 14his-tag fused to nsp8 can be easily removed following TEV-protease cleavage.
-This statement should be modified to state that they have not tested if the tag is removable and the effect on the prep. If the authors did remove the tag, they should show the resulting gels and biochemical activity.

4. The plasmid is not yet deposited on Addgene- do the authors plan to do so when the paper is published? If so, change Our construction has been made available to the entire scientific community through the Addgene plasmid repository (Addgene ID: 165451) upon publication of this manuscript.

5. In the conclusion:
The resulting sample is pure, homogeneous, properly active, and therefore suitable for drug screening and extensive site directed mutagenesis, as well as for functional and structural studies.
-Reword as pure and homogeneous means the same in this context. Properly active is redundant; change to active. Remove extensive. To state that the resulting sample (from the single-day prep) is suitable for structural studies has not been shown. They could say that the one-day prep is ideal for biochemical studies, and while the three-step prep SHOULD be suitable for structural studies BUT that remains to be shown. Otherwise, the conclusion can be construed as misleading.
